# Subgenomic promoter recognition by the norovirus RNA-dependent RNA polymerases

**DOI:** 10.1093/nar/gku1292

**Published:** 2014-12-17

**Authors:** Xiaoyan Lin, Lucy Thorne, Zhinan Jin, Loubna A. Hammad, Serena Li, Jerome Deval, Ian G. Goodfellow, C. Cheng Kao

**Affiliations:** 1 Department of Molecular and Cellular Biochemistry, Indiana University, Bloomington, IN 47405, USA; 2 Division of Virology, Department of Pathology, University of Cambridge, Addenbrookes Hospital, Hills Road CB2 2QQ, UK; 3 Alios BioPharma, Inc., 260 East Grand Avenue South, San Francisco, CA 94080, USA; 4 Laboratory for Biological Mass Spectrometry, Indiana University, Bloomington, IN 47405, USA

## Abstract

The replication enzyme of RNA viruses must preferentially recognize their RNAs in an environment that contains an abundance of cellular RNAs. The factors responsible for specific RNA recognition are not well understood, in part because viral RNA synthesis takes place within enzyme complexes associated with modified cellular membrane compartments. Recombinant RNA-dependent RNA polymerases (RdRps) from the human norovirus and the murine norovirus (MNV) were found to preferentially recognize RNA segments that contain the promoter and a short template sequence for subgenomic RNA synthesis. Both the promoter and template sequence contribute to stable RdRp binding, accurate initiation of the subgenomic RNAs and efficient RNA synthesis. Using a method that combines RNA crosslinking and mass spectrometry, residues near the template channel of the MNV RdRp were found to contact the hairpin RNA motif. Mutations in the hairpin contact site in the MNV RdRp reduced MNV replication and virus production in cells. This work demonstrates that the specific recognition of the norovirus subgenomic promoter is through binding by the viral RdRp.

## INTRODUCTION

Noroviruses are the major causative agents of nonbacterial epidemic gastroenteritis worldwide (1–4). Despite the economic impact, our understanding of the norovirus replication cycle and pathogenicity remain poorly characterized (5). Although several animal systems have been used to study the human norovirus (6–8), they are not robust for mechanistic studies of viral replication. Several breakthroughs in the understanding of norovirus replication have been studies by using the murine norovirus (MNV), which replicates in cultured cells and has been demonstrated to be an excellent model system to study the infection process of the norovirus infection (9–11).

Noroviruses are nonenveloped viruses with a positive-sense, single-stranded, polyadenylated RNA genome in the *Caliciviridae* family. The majority of noroviruses express three conserved open reading frames named ORF1–3 while MNV has an additional ORF4 (12,13). ORF1 encodes a polyprotein which is processed by proteolysis to generate six non-structural (NS) proteins that replicate and transcribe the viral genome. These include NS7, the RNA-dependent RNA polymerase (RdRp) (14). ORF2 and ORF3 encode the major and the minor capsid proteins, respectively. ORF4 of MNV is translated from an alternative reading frame within ORF2 to produce virulence factor 1 that targets innate immune responses (13). The proteins from ORF1 are translated from the norovirus genomic RNA. The proteins from ORF2, ORF3 and ORF4 are translated from one polyadenylated RNA of ca. 2.5 kb in length, the subgenomic RNA (sgRNA) (12,13).

Like all positive-sense RNA viruses, norovirus genome replication occurs via a negative-sense RNA intermediate synthesized by the viral RdRp. The norovirus RdRps initiate RNA synthesis from the nucleotides at the 3′ termini of the genomic positive- and negative-sense RNAs. The positive-sense genomic RNA is synthesized by a protein-primed mechanism, in which a tyrosine residue in the protein primer VPg is used for nucleotide addition (15,16). The negative-sense genomic RNA is initiated using a nucleotide triphosphate as the priming nucleotide, also known as a *de novo* initiation mechanism (17–19).

Subgenomic RNA synthesis by positive-strand RNA viruses has been proposed to occur from either full-length or prematurely-terminated minus-strand RNA (20,21). Morales *et al*. (22) showed that a related member of the *Caliciviridae*, rabbit hemorrhagic disease virus, makes sgRNAs by internal initiation on the minus-strand RNA. In agreement with this model, a highly conserved RNA stem-loop structure upstream of the start site of the sgRNA initiation nucleotide was identified in all caliciviruses. Mutations in this RNA structure will debilitate MNV sgRNA synthesis and suppressor mutations that restored the RNA structure were found to restore MNV infectivity (23,24).

In this study, we demonstrate that chemically synthesized RNAs that contain the putative subgenomic core promoter and a short template derived from the GII.4 human norovirus or MNV can accurately and specifically direct the initiation of RNA synthesis by the respective recombinant RdRps of the two viruses. We have also identified the residues in the MNV RdRp that contact the subgenomic hairpin. Recombinant MNV RdRps with substitutions in the putative contact sites decreased *de novo* initiated RNA synthesis and had defects in the binding of the subgenomic core promoter. In addition, mutations in these residues were either lethal or reduced viral replication when introduced into the MNV infectious clone.

## MATERIALS AND METHODS

### Expression and purification of recombinant MNV RdRp and GII.4 RdRp

The cDNA encoding the MNV RdRp was amplified by polymerase chain reaction (PCR) from the cDNA clone of MNV-1 strain CW1 (GenBank accession number: DQ285629.1) to contain a 5′ NdeI restriction endonuclease cleavage site and a BamHI site at the 3′ terminus. Following restriction enzyme digestion, the cDNA was cloned into the *Escherichia coli* expression vector pET15b (Novagen) to express the recombinant MNV RdRp with a six-histidine tag at the N-terminus. Site-specific mutations in the MNV RdRp were introduced using the Quick-Change site-directed mutagenesis kit (Agilent Technologies). All constructs used in this study were confirmed by sequencing with the BigDye Terminator v3.1 cycle sequencing kit (Applied Biosystems).

Table 1.

**Table 1.** Primer and probes used for qPCR determination of viral genome copies

Primer	MNV genome residues	Sequence
Sense	5028–5046	5′-CCGCAGGAACGCTCAGCAG
Antisense	5137–5156	5′-GGCTGAATGGGGACGGCCTG
TaqMan probe	5962–5076	5′-ATGAGTGATGGCGCA
Fluorophore	FAM	
Quencher	TAMIRA	

*Escherichia coli* Rosetta cells harboring the pET15bMNVRdRp plasmid were grown at 37°C until the optical density at 600 nm reached between 0.6 and 0.8. Isopropyl thiogalactoside (IPTG) was added to a final concentration of 1 mM to induce protein expression for 16–18 h at 16°C. The cells were harvested by centrifugation and resuspended in lysis buffer A (100 mM TrisCl, pH 7.9, 300 mM NaCl, 10% glycerol, 15 mM imidazole, 5 mM β-mercaptoethanol, 0.1% Triton X-100, a protease inhibitor cocktail [Sigma–Aldrich]) and sonicated on ice three times for 15 s each. The lysate was clarified by centrifugation at 11 000 g for 30 min and the supernatant was mixed with Ni-NTA resin according to the manufacturer's instructions (Qiagen). The slurry was packed in a column, washed extensively with lysis buffer containing 30 mM imidazole and eluted with lysis buffer containing 200 mM imidazole. The eluted protein was stored in storage buffer (50 mM TrisCl, pH 7.9, 300 mM NaCl, 20% glycerol, 5 mM Dithiothreitol (DTT)).

Recombinant GII.4 norovirus RdRp was expressed and purified as previously described (19). Briefly, the Top10 *E. coli* cells transformed with pBAD GII.4RdRp plasmid were cultured at 37°C until OD_600_ was around 0.6. l-Arabinose was added to a final concentration of 0.02% to induce the protein expression. The cells were continually grown at 37°C for 5 h and then harvested by centrifugation and suspended in buffer A for cell lysis and purification using the Talon metal affinity resin (Clontech Laboratories) using the manufacturer's instructions. The recombinant protein was washed with lysis buffer containing 20 mM imidazole and eluted with the same buffer that contained 100 mM imidazole. The eluted protein was stored in buffer containing 50 mM 4-(2-hydroxyethyl)-1-piperazineethanesulfonic acid (HEPES) pH 7.9, 150 mM NaCl, 20% glycerol, 5 mM DTT. All recombinant proteins were quantified by using the Bradford assay and the purity assessed using sodium dodecyl sulphate-polyacrylamide gel electrophoresis (SDS-PAGE) stained with Coomassie brilliant blue. The recombinant proteins were stored at −80°C for future use.

### RNA synthesis assay

RdRp assays were performed as previously described by Yi *et al.* with minor modifications (25). All RNAs used in this study, including those with 4-thiouridylate (4-SU) were chemically synthesized (Thermo Scientific). The MNV and GII.4 proscripts were designed according to strains MNV CW1 isolate (DQ285629) nt 5012 to 5059 and GII.4 isolate Hu/GII.4/MD-2004/2004/US (DQ658413) nt 5044 to 5085. Both RNAs contain three nonviral nucleotides (GCG) at their 5′ termini to allow the incorporation of radiolabeled ^32^P-CTP during RNA synthesis *in vitro*. All RNA synthesis reactions were of 20 μl and contained 20 mM sodium glutamate (pH 8.2), 12.5 mM dithiothreitol, 4 mM MgCl_2_, 1 mM MnCl_2_, 0.5% Triton X-100 (v/v), 0.2 mM guanosine triphosphate (GTP), 0.1 mM adenosine triphosphate (ATP) and uridine thriphosphate (UTP), 33.3 nM [α-^32^P]- cytidine triphosphate (CTP) (MP Biomedicals), 50 nM of template RNA and 250 nM of recombinant RdRp unless indicated otherwise. The reaction was incubated at 30°C for 2 h, and then stopped by the addition of ethylenediaminetetraacetic acid (EDTA) (pH 8.0) to a final concentration of 10 mM. RNA products were directly loaded onto a 24% polyacrylamide gel containing 7.5 M urea. The radiolabeled RNA products were visualized and quantified by using a PhosphoImager (Typhoon 9210; Amersham Biosciences) and ImageQuant software.

### Reversible crosslinking peptide fingerprinting analysis (RCAP)

The RCAP assay was carried out as previously described (26). Briefly, the reaction was performed in a 25 μl volume with 2 μM of purified recombinant MNV RdRp and 4 μM of chemically synthesized RNA. Formaldehyde was added to a final concentration of 0.1% (v/v) for 10 min to crosslink the RNA–protein complexes. After quenching the crosslinking with 0.2 M of glycine, sequencing-grade trypsin (Promega) was added at 1:50 (w/w) along with 100 mM NH_4_HCO_3_ (pH 7.8) to digest the sample overnight at 37°C. 3 M LiCl was used to precipitate the RNA–peptide complex followed by two 70% ethanol washes. RNA–peptide crosslinks were reversed by incubating the samples for 1 h at 70°C in a buffer containing 50 mM Tris pH 7.5, 200 mM NaCl, and 0.1% trifluoroacetic acid. The samples were purified and concentrated using a Ziptip column (Millipore) and analyzed by MALDI-ToF mass spectrometry (MS) (Bruker Autoflex III; Agilent Technologies) in positive-ion mode. Assigned peaks all corresponded to within 0.5 Da of the theoretical masses of peptides from MNV RdRp.

### Mapping proscript-RdRp crosslinks using 4SU-substituted RNAs

RdRp crosslinking reactions were performed in clear-bottom 96-well microplates. The reactions were of 25 μl in volume and contained 2 μM purified MNV RdRp and 4 μM of 4SU-labeled RNA in a buffer containing 20 mM HEPES pH 7.5, 4 mM MgCl_2_, 1 mM MnCl_2_ and 1 mM DTT. The samples were transferred to microplates on ice and ultraviolet (UV) crosslinking was performed at 365 nm for 10 min. Trypsin digestion and precipitation of the peptide–RNA complexes were as described above. The pellet was suspended in a buffer containing 50 mM Tris pH 7.5, 200 mM NaCl and digested with RNAse A (1 μg) at 37°C for 30 min. Peptides and peptide–nucleotide conjugates were purified using a C18 column and analyzed by LC–ESI–MS/MS using an nanoliquid chromatography system (AB SCIEX) interfaced to an externally calibrated LTQ Orbitrap hybrid mass spectrometer equipped with a nanospray ion source (Thermo Scientiﬁc). A 4 μl aliquot of the peptide digest dissolved in 12 μl of mobile phase A was washed with buffer A for 5 min at a flow rate of 5 μl min^−1^ using a reversed-phase C18 trap column made from a 15 mm × 100 μm integrafrit column and packed with Magic C18 resin (200 Å pore size, 5 μm particle size; Bruker-Michrom). The desalted peptides were then separated with Buffer B gradient using a reversed-phase C18 column (Magic C18, 100 Å, 5 μm particle size, Bruker-Michrom) packed in a 150 mm × 75 μm, 15 μm tip (New Objective) and analyzed in positive ion mode. Buffer A consisted of 0.1% formic acid in acetonitrile:water (3%:97%, v/v) and Buffer B consisted of 0.1% formic acid in acetonitrile:water (97%:3%, v/v). The flow rate was 300 nl min^−1^ with the following gradient: 3% B from 0–1 min, ramp to 10% B from 1–9 min, ramp to 30% B from 9–28 min, ramp to 40% B from 28–31 min, ramp to 80% B from 31–35 min, hold at 90% B from 36–43 min, ramp to 3% B from 43–44 min, then re-equilibrate the column at 3% B for 14 min (58 min total run time). The mass spectrometer was operated in an automated data dependent mode, alternating between an FT-MS scan in the Orbitrap and five collision-induced dissociation (CID) scans in the linear ion trap. The Orbitrap monitored precursor ions from *m/z* 300 to *m/z* 2000 at 15 000 resolving power. The five most intense precursor ions in each scan were sequentially isolated in the linear trap with a 2 *m/z* isolation width and activated at 35% normalized collision energy. The cycle was continuously repeated throughout the entire separation with the dynamic exclusion set to 45 s with a repeat count of 2. Identification of peptides covalently attached to 4SU was performed using the Proteome Discoverer™ software by searching the raw LC–MS/MS data using SEQUEST^®^ against the protein sequence and allowing for variable modifications corresponding to possible 4SU structures attached to the protein.

### Fluorescence polarization assay

All assays were performed in 20 μl reactions. 0.1 μM of the FL-MNV-proscript was mixed with MNV RdRp at various concentrations in the reaction buffer containing 10 mM Tris–HCl pH 8.0, 30 mM NaCl, 1% glycerol and 0.5 mM 2-mercaptoethanol. A Victor^3^V 1420 multi-label counter (Perkin Elmer) was used to read fluorescence polarization data from the plates. The excitation and emission wavelengths were set at 485 and 535 nm, respectively. The data were plotted against MNV RdRp concentration and the data were fit to a hyperbola (*Y* = min + (max − min) × *S*/(*S* + *K*_d_)).

### Differential scanning fluorimetry

Differential scanning fluorimetry (DSF) was used to measure the thermodenaturation profiles of proteins. DSF used a Stratagene MX3005P real-time PCR machine. Each sample was prepared in a total volume of 25 μl that contained a 5× final concentration of SYPRO Orange (Molecular Probes) in buffer containing 20 mM sodium glutamate (pH 8.2), 12.5 mM dithiothreitol, 4 mM MgCl_2_ and 1 mM MnCl_2_. Recombinant MNV RdRp or mutant R411A was mixed with Mps at a 1:2 molar ratio. All reactions used a ramp rate of 0.5°C/min and a temperature range from 25 to 75°C. The results were analyzed and plotted using GraphPad Prism.

### Cells and plasmid constructs

The murine microglial BV-2 cell line was maintained in Dulbecco's modified Eagle's medium (DMEM) supplemented with 10% fetal calf serum (FCS), 2 mM l-glutamine, 0.075% sodium bicarbonate, 100 units/ml penicillin and 100 μg/ml streptomycin (27). BHK cells engineered to express T7 RNA polymerase (BSR-T7 cells, obtained from Karl-Klaus Conzelmann, Ludwig Maximilian University, Munich, Germany) were maintained in DMEM containing 10% FCS, penicillin (100 SI units/ml), streptomycin (100 μg/ml) and 0.5 mg/ml G418. The cDNA clone pT7: MNV3′ RZ, containing the WT MNV-1 genome under the control of a truncated T7 polymerase promoter, had been previously constructed (28).

### Virus recovery and growth curve analysis

The R411A, R411K, R416A and R411/416AA mutations were introduced into the MNV-1 infectious clone (pT7:MNV 3′RZ) by overlapping mutagenic PCR. Virus was rescued from cDNA clones using the reverse genetics system based on recombinant Fowlpox expressing T7 RNA polymerase, previously described in (24). Briefly, BSR-T7 cells were infected with recombinant Fowlpox virus expressing T7 RNA polymerase at an MOI of approximately 0.5 pfu/cell, prior to transfection with 1 μg of each cDNA clone using Lipofectamine 2000 (Invitrogen). Forty-eight hours post-transfection (hpt), cells were freeze-thawed at −80°C to release virus particles, and titered by limiting dilution to obtain the 50% tissue culture infectious dose (TCID_50_) titrations were performed on BV-2 cells using 10-fold serial dilutions. The viral TCID_50_ was determined by scoring for signs of cytopathic effect at 5 days post-infection by visual inspection. Western blot for the viral RdRp used rabbit anti-MNV NS7 serum (non-commercial Ab) and mouse anti-GAPDH (Applied Biosystems) as a loading control. HRPO-conjugated primary and secondary goat antibodies recognizing rabbit or mouse immunoglobulins were detected by chemiluminescence (ECL, Amersham, GE Healthcare). Passage 1 stocks of the viable viruses were generated by inoculating BV-2 cells (seeded at a density of 1 × 10^5^ in six-well plates) with 700 μl of recovered virus for 1 h. RNA was extracted from the stocks using the GenElute™ System (Sigma–Aldrich) for RT-PCR amplification and verification of the mutations by DNA sequencing.

### RNA secondary structure prediction and protein model generation

All RNAs secondary structures in this study were predicted by using the program Mfold (29). Molecular graphics and analyses of the MNV RdRp (PDB: 3QID) were performed with the University of California San Francisco Chimera Package (www.cgl.ucsf.edu/chimera).

## RESULTS

### Norovirus RdRps can specifically synthesize RNA from their subgenomic core promoters

**Figure 1.**
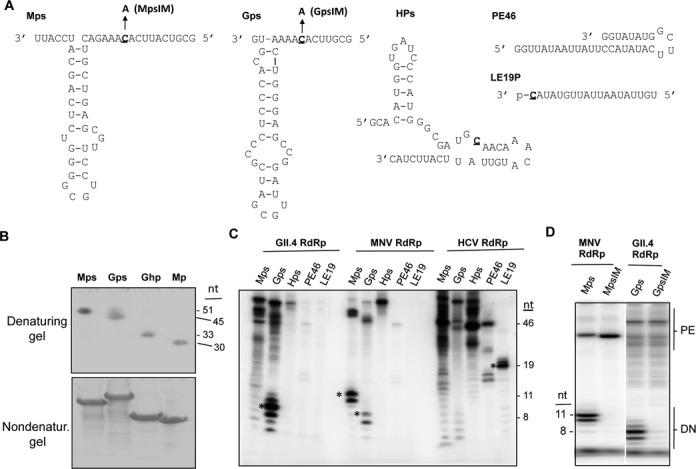
Recombinant norovirus RdRps can initiate RNA synthesis from their proscripts. (**A**) Sequences and predicted RNA structures of five RNAs tested for RNA synthesis by recombinant RdRps. The names for the RNAs are in bold. Where present, the template initiation residue is underlined. Mutant MpsIM and GpsIM is a version of the Mps and Gps with the initiation cytidylate substituted with an adenylate. Mps; predicted secondary structure of the MNV CW1 isolate (DQ285629), nt 5012 to 5059. Gps; the sequence and predicted secondary structure of the GII.4 isolate Hu/GII.4/MD-2004/2004/US (DQ658413), nt 5044 to 5085. Hps; predicted secondary structure of the HEV genotype 3 Kernow C1 isolate (JQ679013), nt 5317 to 5361. (**B**) The RNAs used for RNA synthesis exist in one predominant conformation in solution. The upper gel image shows the RNAs used in the analysis in this manuscript separated in a 15% polyacrylamide gel amended with 7.5 M urea. The bottom gel image contains RNAs separated in a 15% polyacrylamide gel that lacks urea. The RNAs were stained with toluidine blue. (**C**) RNA products made by the RdRps from the GII.4 human norovirus, MNV, and the HCV. Products correctly initiated by a *de novo* mechanism and terminated after the 3′ terminus of the template are identified by asterisks. The gel image was obtained using a Phosphorimager of a 24% polyacrylamide gel that contains 7.5 M urea. The lengths of the de novo initiated RNAs were derived from previous analyses where the MNV RdRp products were electrophoresed along with products of known lengths. (**D**) Proscripts with substitutions of the *T* + 1 cytidylate are unable to direct *de novo* initiation of RNA products by the MNV and the GII.4 RdRps. The products of primer extension (PE) and *de novo* initiation (DN) are shown. The RNAs that are correctly initiated and terminated from Mps and Gps are, respectively, 11- and 8-nt.

We hypothesized that the norovirus RdRp is the component in the replicase complex that recognizes core promoters for sgRNA viral RNA synthesis. Recombinant viral RdRps can typically use a wide variety of RNA templates for RNA synthesis and hence, have not been thought to specifically recognize viral promoter elements except for the initiation nucleotide (30,31). Five RNA promoter/templates were designed to examine specificity for RNA synthesis (Figure 1A). Three of the RNAs contained viral subgenomic core promoters followed by short template sequences. For brevity, these RNAs are referred to as ‘proscripts’. The MNV, human norovirus GII.4, and the Hepatitis E virus (HEV) proscripts are, respectively, named Mps, Gps and Hps (Figure 1A). The remaining two RNAs, PE19 and PE46, do not contain promoter sequences, but were previously used for, respectively, *de novo* initiated and primer-dependent RNA synthesis by the Hepatitis C virus RdRp (32).

The structures of the MNV and GII.4 subgenomic hairpin are conserved and the base pairing in the MNV hairpin has been shown to be required for efficient MNV infection (24). The computer program Mfold also shows that the hairpin structure is the thermodynamically most stable in the context of the proscript RNAs (data not shown). However, to determine whether the MNV and GII.4 proscripts will form one predominant RNA structure in solution, we analyzed the RNAs using denaturing and nondenaturing polyacrylamide gel electrophoresis. This analysis was previously used to characterize the suitability of the RNAs for NMR spectroscopy (33,34). In the denaturing gels, all of the RNAs migrated at their expected lengths (Figure 1B). In the nondenaturing gels, the RNAs also formed one band, suggesting that Mps and Gps form one predominant RNA structure in solution (Figure 1B). RNAs Mhp and Ghp that contain the hairpin structures also formed one predominant structure in solution (Figure 1B).

Highly purified recombinant RdRps from the MNV, the GII.4 human norovirus, and HCV were tested for their abilities to use the five RNAs for RNA synthesis. Accurate initiation of RNA synthesis from the nucleotides that direct sgRNA synthesis during infection should yield products that are distinguishable in length. Mps, Gps and Hps should, respectively, yield RNAs of 11-nt, 8-nt and 24-nt. LE19P directs the synthesis of a *de novo* initiated 19-nt product. It also contains a 3′ terminal puromycin that blocks primer extension. PE46 forms a stable self-annealed RNA whose 3′ terminus can be extended by several RdRps to form a ca. 46-nt RNA product (32).

Consistent with previous results, the HCV RdRp formed primer-extension products from PE46 that were 46-nt in length as well as a 19-nt *de novo*-initiated RNA from LE19P (Figure 1C). The HCV RdRp did not produce significant amounts of *de novo* initiated products from the three proscripts. Instead, the products generated were of lengths longer than the proscripts (Figure 1C). These products are likely due to primer extension of the RNAs when the 3′ termini of the proscripts annealed with the single-stranded regions of the proscript (Figure 1C). Thus, all five RNAs are capable of directing some form of RNA synthesis by the HCV RdRp, although the HCV RdRp did not preferentially use the initiation template nucleotides of the three proscripts.

The MNV RdRp produced major products of ca. 50-, 11- and 10-nt from Mps. The ca. 50-nt product likely arose by primer extension. The 11-nt product likely initiated from the initiation cytidylate in the template (to be named *T* + 1 cytidylate) and terminated at the end of the template sequence in the proscript. The 10-nt product likely arose from correct *de novo* initiation from the *T* + 1 cytidylate but terminated one nucleotide before the RdRp reached the final 3′ nucleotide of the template RNA. Premature termination of RNA synthesis has been reported for several recombinant RdRps *in vitro* (35,36). To confirm that the MNV RdRp can accurately initiate RNA synthesis from the *T* + 1 cytidylate, we used proscript MpsIM, which has the same sequence as Mps except for an adenylate substitution at the *T* + 1 position. MpsIM did not direct the synthesis of either the 11- or 10-nt product, but did generate primer extension products longer than the input template (Figure 1D). This result demonstrates that the MNV RdRp can accurately initiate RNA synthesis from Mps. Interestingly, in multiple trials the primer-extended product was more abundant with MpsIM than with Mps, suggesting that the lack of *de novo* initiated RNA synthesis enabled the RdRp to engage in primer-dependent RNA synthesis.

The MNV RdRp produced only a low abundance of the 8-nt *de novo* initiated product from the GII.4 proscript, Gps. A 7-nt product which initiated correctly, but terminated 1-nt short of the 3′ of the template, was also produced (Figure 1C). With Hps, only the primer-extended products were observed. With PE46, the MNV RdRp produced only primer-extended products while LE19P did not direct RNA synthesis (Figure 1C). Altogether, these results show that the MNV RdRp preferentially used its own proscript to direct RNA synthesis.

The GII.4 RdRp also exhibited accurate *de novo* initiation from its own proscript *in vitro* (Figure 1C). An RNA with a substitution in the initiation cytidylate of Gps named GpsIM failed to produce *de novo* initiated RNA (Figure 1D). The MNV proscript also failed to direct *de novo* initiated RNA products, although some primer-extension products were made. As with the MNV RdRp, the GII.4 RdRp also produced only low amounts of products from Hps, PE46 and LE19P. Similar to the MNV RdRp, the GII.4 RdRp has a strong preference for its cognate proscript RNA for RNA synthesis.

### Elements in Mps that contribute to recognition by the MNV RdRp

**Figure 2.**
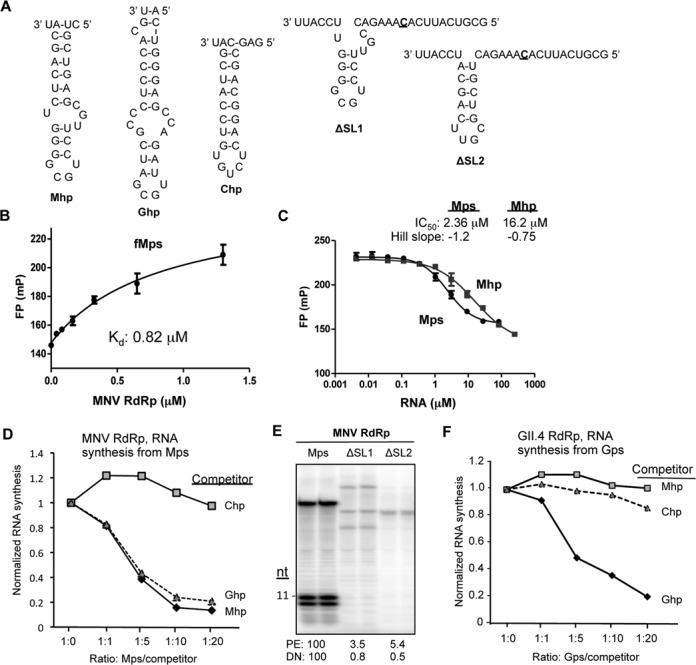
The hairpin within the MNV subgenomic proscript is required for binding by RdRp and to direct RNA synthesis. (**A**) RNAs use in this analysis. The names of the RNAs are in bold. ΔSL1 and ΔSL2 have deletions in the hairpin of Mps, but the template sequences are unchanged. The template initiation cytidylate is in bold and underlined. (**B**) Fluorescence polarization results assessing the affinity of MNV RdRp for fMps. RNA fMps was chemically synthesized to contain a 5′ 6-carboxyfluorescein (6-FAM) attached to Mps. The binding isotherm is shown and all data points were assessed in triplicate. The results are reproducible with four independent assays. (**C**) A competition assay assessing the inhibitory activities of Mps and Mhp on MNV RdRp binding to fMps. The effective inhibitory concentration (IC_50_) and the Hill slope for the competitor RNAs, Mps and Mhp, are shown above the graph. IC_50_ values were derived using the algorithm developed by Nikolovska-Coleska *et al.* (61). (**D**) The hairpin in Mps and the Gps can bind to the MNV RdRp. The graph shows the results from an RNA synthesis assay performed with the MNV RdRp in the presence of increasing concentrations of three competitor RNAs, Chp, Ghp and Mhp. Comparable results were obtained in three independent experiments. (**E**) Deletions in the hairpin within Mps severely reduced *de novo* initiated RNA synthesis. The gel image was from an RNA synthesis assay with the template RNAs all being at 0.05 μM. (**F**) The GII.4 norovirus RdRp can specifically recognize the hairpin in the GII.4 norovirus proscript. The graph shows the results from RNA synthesis assay performed with the MNV RdRp in the presence of increasing concentrations of three competitor RNAs, Chp, Ghp and Mhp.

To determine the affinity of the MNV RdRp for its proscript. Mps was synthesized to contain a fluorophore at its 5′ terminus, resulting in RNA fMps. fMps directed product synthesis by the MNV RdRp in a manner indistinguishable from Mps (data not shown). Fluorescence polarization assays generated the binding isotherms that were fitted to a one site binding model. The MNV RdRp had a *K*_d_ of between 0.5 and 1 μM for fMps (Figure 2B).

We sought to determine whether Mph, the hairpin within Mps, confers stable binding to Mps. fMps pre-incubated with the MNV RdRp was challenged with increasing concentration of Mps or Mph (Figure 2A). The resulting fluorescence polarization data were plotted against the RNA concentration and the data were fitted to a Hill equation, yielding the values of IC_50_ for Mps and Mhp of 2.36 and 16.2 μM, respectively (Figure 2C). The *K*_i_ values calculated from these IC_50_ values were 0.68 μM for Mps and 6.3 μM for Mhp. These results suggest that while the hairpin portion of the Mps binds the MNV RdRp, the template sequence contributes to stable binding.

Next we assessed whether the hairpin in Mps is required for RNA synthesis by the MNV RdRp. RNA synthesis reactions programmed with the MNV RdRp and Mps were challenged with increasing concentrations of either Mph, Gph (the hairpin from the GII.4 proscript), or Chp (the hairpin from the Chikungunya virus proscript) (Figure 2A). RNA synthesis from Mps was not affected by the addition of Chp (Figure 2C). However, both Ghp and Mhp were effective inhibitors of RNA synthesis. These results suggest that the MNV RdRp preferentially recognizes the norovirus subgenomic hairpin, but it could not distinguish between Mhp and Ghp under the conditions used *in vitro* (Figure 2D).

To further determine the contribution of the hairpin in Mps to recognition by the MNV RdRp, RNAs with deletions of portions of the Mps hairpin named ΔSL1 and ΔSL2 were tested (Figure 2A). Both retained the initiation sequence for RNA synthesis *in vitro*, but neither could direct *de novo* initiated RNA synthesis (Figure 2E). The amount of the primer-extension products from ΔSL1 and ΔSL1 were also sharply decreased relative to those from Mps, suggesting that the hairpin structure and/or sequence is necessary for recognition by the MNV RdRp (Figure 2E). Both the template and the hairpin structure within the Mps are required for efficient RNA synthesis.

RNA synthesis by the GII.4 RdRp was also tested in the presence of increasing concentrations of competitor hairpin RNAs. Ghp inhibited RNA synthesis by the GII.4 RdRp from proscript Gps in a concentration-dependent manner (Figure 2F). Notably, the degree of inhibition was similar to the inhibition of the MNV RdRp by Mph. Interestingly, both the Mhp and the Chp were poor inhibitors of RNA synthesis by the GII.4 RdRp from Gps. This demonstrates that the GII.4 RdRp has higher specificity for the hairpin in the GII.4 proscript than does the MNV RdRp for its cognate subgenomic hairpin. This higher specificity by the GII.4 RdRp for the Gps hairpin likely accounts for its decreased *de novo* initiated RNA products from Mps (Figure 1B).

### The role of the initiation sequence in viral RNA synthesis

**Figure 3.**
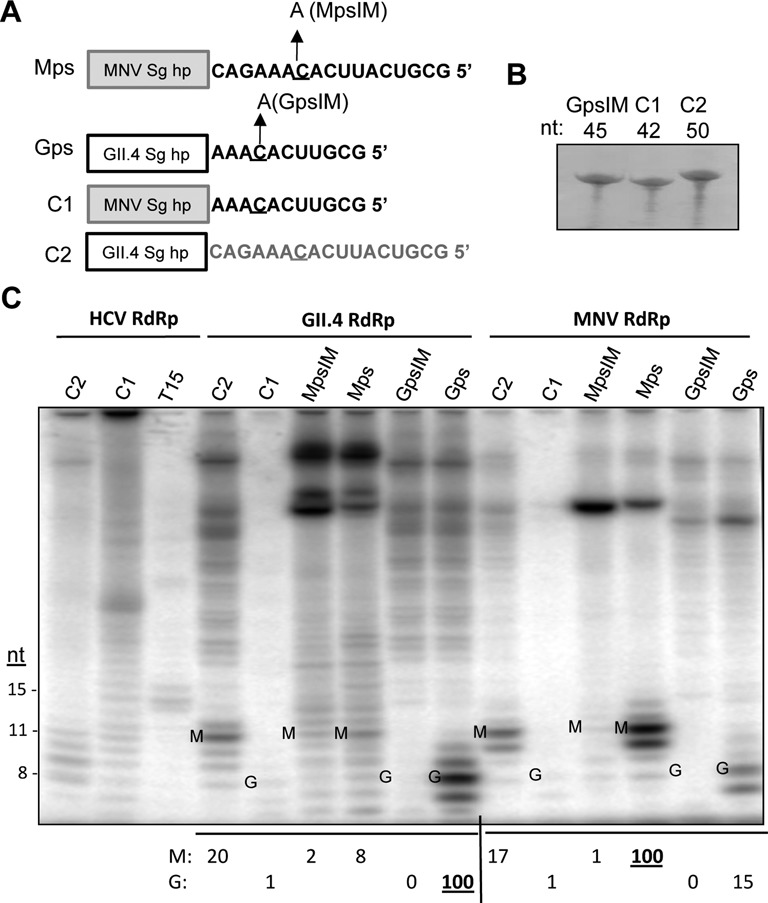
The single-stranded RNA sequence in Mps and Gps affects norovirus RNA-dependent RNA synthesis. (**A**) Schematics of the chimeric RNAs examine in this assay. The box denotes the hairpin structure while the single-stranded RNA sequences are shown in their entirety. (**B**) Gel image of the chemically synthesized RNAs C1 and C2 and the GpsIM. The RNAs were resolved in a 24% polyacrylamide gel amended with 7.5 M urea. The RNAs were visualized by staining with methylene blue. (**C**) RNA products by recombinant viral RdRps from the C1 and C2 RNAs as well as controls. The gel image contains products from the viral RNA synthesis assay. The lengths of the *de novo* initiation products are identified to the left side of the gel image. Correctly *de novo* initiated and terminated products from the MNV template sequence are labeled with a M. Correctly *de novo* initiated and terminated products from the GII.4 template sequence are labeled with a G. The amounts of synthesized RNA are quantified, normalized to that of the wild-type proscript, and shown below the gel image. Products of the GII.4 and MNV RdRps are all normalized to the amounts produced by the RdRp from its homologous proscript (denoted as an underlined 100).

Both viral replicases and DNA-dependent RNA polymerases recognize the nucleotides that encompass the initiation nucleotide (36–38). To determine whether the single-stranded sequence in the proscripts contributes to RNA synthesis directed by the norovirus RdRps, two chimeric RNAs that swapped the hairpins of the Mps and Gps were made and tested for their ability to function *in vitro* (Figure 3A). Chimera 1 (C1) that has the hairpin from MNV proscript and the single-stranded sequence from GII.4 proscript was poor in directing RNA synthesis by the HCV, GII.4 and MNV RdRps, despite the RNA being the correct length and concentration (Figure 3B). RNA folding programs predicted that C1 would form the expected hairpin and single-stranded sequence (data not shown). However, given the poor synthesis, interpretation of the results from the C1 RNA should be kept at a minimum.

Chimera 2 (C2), which has the single-stranded sequence from MNV and the hairpin from GII.4, directed the GII.4 RdRp to produce 20% of the *de novo* initiated product when compared to Gps. This indicates that the GII.4 RdRp strongly prefers its own single-stranded sequence. The GII.4 RdRp also produced more *de novo* initiated RNA from C2 when compared to Mps (20 versus 8; Figure 3C), consistent with the idea that the cognate hairpin could promote *de nov*o initiated RNA synthesis.

With the MNV RdRp, C2 directed only 17% of the product relative to the amount from Mps. Given that Mps and C2 have identical single-stranded sequences, the reduced level of RNA synthesis demonstrates that the MNV Sg hairpin is needed for efficient RNA synthesis (Figure 2D and E). C2 directed the MNV RdRp to produce similar amounts of *de novo* initiated RNA when compared to Gps (17 versus 15; Figure 3C). Given that C2 and Gps share the same hairpin sequence but differ in their single-stranded sequences, the MNV RdRp has more flexible recognition of the single-stranded sequence than does the GII.4 RdRp. Thus, although the MNV RdRp preferentially recognizes its own subgenomic proscript, it has lower specificity for both the hairpin and the single-stranded RNA sequence when compared to the GII.4 RdRp. This lower specificity likely accounts for the higher level of promiscuous RNA synthesis from the Gps by the MNV RdRp.

### Identification of the regions of the MNV RdRp that contact the subgenomic proscript

**Figure 4.**
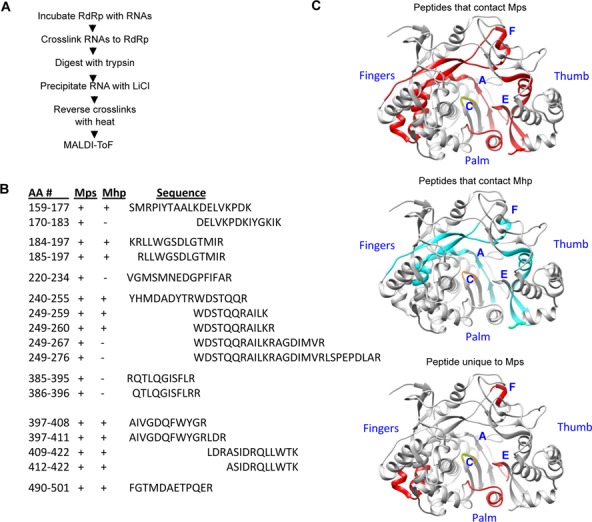
Regions of the MNV RdRp that contact Mps and Mhp. (**A**) Schema of the RCAP protocol used to map RdRp-RNA contacts. (**B**) MALDI-ToF identified peptides from the MNV RdRp that contacted Mps or Mhp. The peptides that contacted the Mps and Mhp are in the columns labeled ‘Mps’ and ‘Mph’, respectively. A plus (+) symbol indicates that the peptide was observed. The (−) symbol indicates that the peptide was not observed. The sequences of the peptides are listed in the standard one-letter amino acid codes, and the peptides are aligned to show overlaps in the sequences. (**C**) Regions in the MNV RdRp that contacted Mps (red) or Mhp (blue) or only the Mps (red). The structure of the MNV RdRp was derived from PDB 3QID and the model was made with the software Chimera (UC San Francisco). The approximate locations of RdRp motifs A, C, E and F in the MNV RdRp are labeled in blue.

We sought to identify the specific amino acids in the norovirus RdRp that contact the subgenomic proscript. Given that the MNV system offers greater flexibility for genetic manipulation than the human norovirus, we focused this mapping effort on the MNV RdRp despite its reduced specificity for its proscript. The mapping studies used the RCAP protocol, which combines reversible crosslinking and peptide mass fingerprinting to identify peptide sequences within the RdRp that can crosslink to the viral RNA (Figure 4A). Briefly, the RNAs and RdRp were crosslinked with formaldehyde, followed by extensive digestion of the complex with trypsin. The RNAs and peptides associated with RNA were precipitated with LiCl and the crosslinks reversed by heating. MALDI-ToF/MS was then used to identify the RNA-associated peptides. Control reactions that contained the MNV RdRp treated with formaldehyde in the absence of RNA, or the RdRp-RNA complex that were not crosslinked with formaldehyde did not yield signals above background. However, 17 peptides derived from the MNV RdRp were identified to be associated with Mps, and 11 with Mhp (Figure 4B and Supplementary Figure S1). Within each data set, several peptides had overlapping sequences due to missed cleavages by trypsin, which is expected to occur when an arginine or lysine residue is crosslinked to the RNA. Eight of the 17 MNV RdRp peptides identified to associate with Mps, only 8 represent unique regions in the RdRp. In addition, all the peptides identified as associated with Mhp were also present in reactions performed with the MNV RdRp and Mps. This should be expected, given that Mps contains the hairpin that is Mph. To visualize the locations of these peptides, we mapped these peptides on the MNV-1 RdRp crystral structure (PDB: 3QID) (39) (Figure 4C). Notably, the majority of the cross-linked peptides are located in the central cavity of the MNV RdRp, where RNA synthesis occurs.

Peptides associated with Mps and Mhp were located near conserved motifs in the MNV RdRp (Figure 4C top and middle panels). Peptides containing residues 159–177 overlapped with motif F1 and motif F2 in the fingers subdomain of the RdRp that is known to interact with the RNA and the substrate nucleotide (40,41). Peptides containing residues 240–255 overlapped with motif A, which contains aspartates that coordinate divalent metal ions needed to bind NTPs and catalyze phosphodiester bond formation (40,41). Peptides containing residues 397–422 were near Motif E, which connects the fingers and palm subdomains and has been proposed to be a part of the platform for *de novo* initiation (42–44).

The six MNV RdRp peptides associated with Mps but not Mhp likely contact the single-stranded sequence in Mps (Figure 4C bottom). Amino acid residues 177–183 are adjacent to motif F and residues 220–234 and 267–276 are near Motif A. Residues 385–396 are overlapped motif E, which may represent the location in the MNV RdRp that contacts both the junction of the subgenomic hairpin and the single-stranded sequence.

### Identification of residues in the MNV RdRp that contact the core promoter hairpin

**Figure 5.**
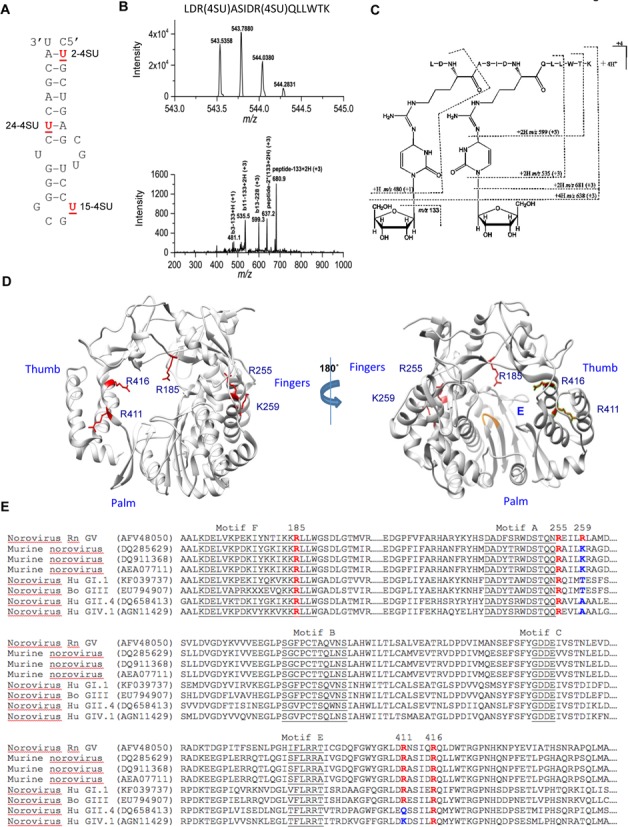
Residues of the MNV RdRp that contact 4-thiouridylate (4SU) within the Mhp. (**A**) Locations of the 4-SU within the chemically synthesized Mhp RNAs. The names of the RNAs with each of the 4-SU residue are in bold. All samples were processed by a UV crosslinking protocol that included exhaustive digestion of the RNAs that should leave only the 4-SU nucleotide covalently attached to peptides. (**B**) Mass spectrum (MS) and collision-induced dissociation (CID) spectrum of the peptide ion [LDR(4SU)ASIDR(4SU)QLLWTK- S_2_]^4+^ obtained on a Thermo LTQ-Orbitrap instrument. (Top) High-resolution mass spectrum (MS) of the peptide ion LDR(4SU)ASIDR(4SU)QLLWTK- S_2_]^4+^ (RdRp residues 409–422) from the reaction with 15-2SU substituted RNA. The measured monoisotopic mass (5 ppm mass error) is at *m/z* 543.5358 (4+). (Bottom) CID spectrum of this peptide ion at *m/z* 543.5. The CID fragment ion assignments are illustrated with the dashed lines in (C) structure. (**C**) Proposed structure and collision induced dissociation (CID) fragments (dashed lines) of the doubly-substituted 4SU peptide. The most common loss upon fragmentation is that of the ribose sugar (*m/z* 133) from either the parent ion or its ‘b ion’ peptide fragments. (**D**) A summary of the locations of the residues in the MNV RdRp that were identified to contact the three 4-SU-modified RNAs. The side chains of the residues are shown in red. (**E**) Alignments of the sequences of the norovirus RdRps that are relevant to the residues found in the MNV RdRp which contacted different regions of Mhp. All sequences are shown with their Genbank accession number. The residues in specific motifs conserved in viral RdRps are underlined. The residues identified to contact the 4SU-modified MNV are in red, as are the comparable residues in other norovirus RdRps. Where there are amino acid substitutions in the key residues, the changes are shown in blue.

To confirm the identities of the MNV RdRp peptides that contact the subgenomic hairpin, we used high efficiency photoactivatable crosslinking between 4-thio-uracil (4SU) analogs and amino acids (45). Three versions of Mhp were chemically synthesized to each contain 4SU at residues 2, 15 or 24 to generate RNAs named, respectively, 2-4SU, 15-4SU, and 24-4SU (Figure 5A). *In vitro* RNA synthesis assays confirmed that the three RNAs inhibited RNA synthesis from Mps to the same degree as the unmodified Mhp (Supplementary Figure S2). Following crosslinking to MNV RdRp and trypsin digestion, the peptide-RNA complexes were precipitated with LiCl. The RNAs in the complexes were then thoroughly digested to release nucleotides from the peptides, leaving only the crosslinked nucleotide. The identities of the cross-linked peptides were then determined by high-resolution and collision induced dissociation (CID) mass spectra using the LTQ-Orbitrap instrument. Kramer *et al.* have shown that crosslinking of proteins and 4SU substituted RNAs could undergo the loss of H_2_S, the loss of the double bond in 4SU, and the loss of phosphates from either or both the 3′ or the 5′ positions (45). All of these structural possibilities were considered during analysis of the Orbitrap mass spectra for the crosslinking reactions between the MNV RdRp protein and the modified RNAs. Three different peptides having one or more 4SU molecules attached to either arginine or lysine residues were identified from the reactions of RdRp with the modified RNAs. The sequence LDRASIDRQLLWTK from residues 409–422 was identified with certainty to bind 15-4SU (Figure 5B). CID data indicated that 15-4SU crosslinked to R411 and R416 side chains and resulted in the loss of its sulfur atom as shown in Figure 5C. Two additional peptides KRLLWGSDLGTMIR (residues 184–197; crosslinked arginine underlined) and WDSTQQRAILK (residues 249–259, crosslinked arginine and lysine underlined) were identified to bind 24-4SU and 2-4SU, respectively (Supplementary Figure S3). However, some signals for both peptides were also observed with 2-SU and 24-4SU, suggesting that the bulged region in the hairpin and the base of the hairpin RNA could contact the RdRp at two different locations. The three 4SU cross-linked peptides identified here were also identified in the RCAP assay discussed previously (Figure 4). The locations of the five cross-linked residues are shown in the model of the MNV RdRp in Figure 5D.

### Functional characterization of the subgenomic promoter hairpin contact sites in the MNV RdRp

To assess the functional relevance of these residues that contact the Mhp, we examined their degree of sequence conservation in the RdRps from multiple noroviruses. Residues R185, R255, and R416 are highly conserved among noroviruses while K259 and R411 were conserved only in murine noroviruses (Figure 5E). We reasoned that the highly conserved residues are more likely to be essential for RNA synthesis while the less conserved amino acids are more likely to be involved in species-specific recognition of the RNA.

**Figure 6.**
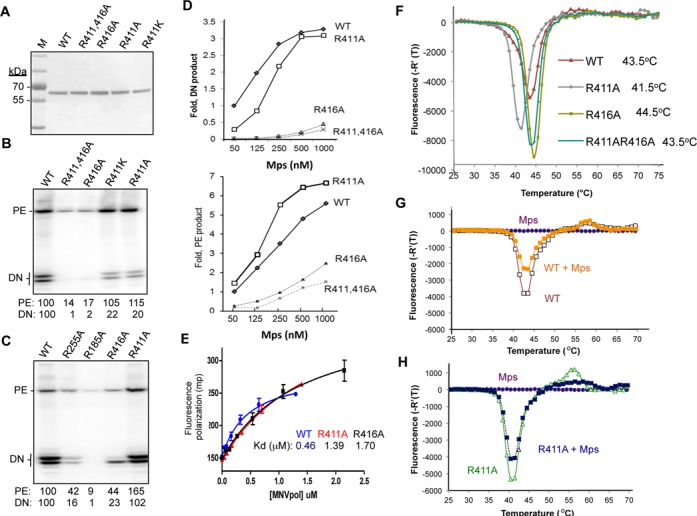
Functional characterization of the effects of mutations in the MNV RdRp for RNA synthesis, binding to the Mps and their thermodenaturation profiles. (**A**) Purified recombinant proteins used in the analysis. The image is a SDS-PAGE stained with Coomassie blue. The molecular weight marker (M) is from Pierce. R411AR416A denotes that the recombinant RdRp contained alanine substitutions at both R411 and R416. (**B**) RNAs synthesized by the WT and mutant MNV RdRps. The concentration of WT or mutated RdRps is 250 nM and the Mps is at 50 nM in each reaction. The products of primer extension (PE) and *de novo* initiation (DN) are shown. The DN products are of 11- and 10-nt in lengths. The amount of RNAs normalized to that of the WT were quantified and shown below the gel image. (**C**) RNA synthesized by the WT and mutant MNV RdRps. The concentration of the WT or mutant RdRps is 1 μM and the Mps is at 50 nM in each reaction. (**D**) Increasing template Mps concentration was able to restore the amount of *de novo*-initiated RNA synthesis by R411A RdRp (upper graph). Interestingly, the relative amounts of primer-extension products were not corrected for the R411A mutant. (**E**) Binding isotherms for Mps by the WT, R411 and R416 RdRps. The dissociation constants derived from the binding isotherms are 0.5, 1.4 and 1.7 μM for the WT MNV, the R411A and R416A mutants. The data were derived from fluorescent polarization assays performed with a 6-FAM-labeled Mps and recombinant RdRps in the absence and presence of Mps. (**F**) The thermodenaturation profile of WT, R411A, R416A and R411AR416A RdRps. The upper panel shows a comparison of the thermodenaturation profiles of the WT MNV RdRp with R411A, R416A, R411AR416A mutants. The R411A RdRp has a *T*_mapp_ 2°C lower than that of the WT RdRp; R416A mutatns has a *T*_mapp_ 1°C higher than that of the WT RdRp; R411AR416A has the same *T*_mapp_ as that of WT RdRp. (**G**) The thermodenaturation profiles of WT RdRp without and with Mps. (**H**) Thermodenaturation profiles of R411A RdRp without and with Mps. The assay was performed with a 1:2 molar ratio of RdRp to RNA.

To determine whether the putative RNA-interacting residues affect RdRp activity, recombinant proteins containing substitutions at these residues were tested for RNA synthesis using Mps (Figure 6A). MNV RdRps with alanine substitutions at the highly conserved R185 and R255 exhibit severely reduced RNA *de novo* initiated RNA synthesis and produced only a fraction of the primer product using Mps as the template (Figures 4 and 6B, C). A mutant RdRp with a substitution at the less conserved K259 had no obvious defect in either *de novo* initiated RNA synthesis or primer extension (Figure 4).

Substitutions at residues R411 and R416 had more complex effects on RNA synthesis. R411 is conserved only in MNV and is located near motif E. When substituted with a lysine or an alanine, the resulting proteins, R411K and R411A, produced ∼5-fold lower *de novo* initiation RNA product than did the WT MNV RdRp (Figure 6B). Interestingly, the abundance of primer extension products made by R411K or R411A was comparable or slightly higher than those produced by the wild-type MNV RdRp. Mutant R416A was severely reduced for both primer extension and *de novo* initiation (Figure 6B). When the concentration of the WT or the mutated RdRps was increased, R411A restored the *de novo* initiation activity to a level similar to that of the WT RdRp. Mutant R416A did produce more products when tested at higher concentrations, but both primer extension and *de novo* initiation remained sharply reduced compared to that of WT RdRp. Surprisingly, of the two major *de novo* initiated RNA products made by R416A, the product prematurely terminated by 1-nt was more abundant than the full-length product (Figure 6C). Recombinant RdRps that are affected in the recognition of the template-nascent RNA duplex have previously been shown to increase premature termination (46). Quantification of the RNAs synthesized by R411A, R416A or a mutant with substitutions at both R411 and R416 are shown in Figure 6D. These results indicate that both R411 and R416 that contact the MNV subgenomic hairpin affect *de novo* initiated RNA synthesis and confer specific recognition of the promoter sequence.

### Residues R411 and R416 contribute to MNV RdRp binding to the subgenomic proscript

A fluorescence polarization assay was used to determine the dissociation constant for fMps binding by R411A and R416A. The mutant R411A had a modest ca. 3-fold decrease in the dissociation constant for Mps when compared to the WT RdRp. The binding of mutant R416A to Mps was also modestly affected at a level similar to that of R411A (Figure 6E). These results complement those from the RNA synthesis assays and confirm that residues R411 and R416 in the MNV RdRp interact with Mps.

The observed defect in RNA binding exhibited by R411A and R416A could be due to a change in the RdRp conformation needed for proscript binding or an effect on the affinity for the proscript. To examine whether RdRp conformation was affected, we used differential scanning fluorimetry (DSF) to measure the thermodenaturation profile of the WT and mutant RdRps. This assay measures the binding of the dye SYPRO orange by hydrophobic regions of proteins that will become increasingly exposed with temperature (47). Bound SYPRO orange will fluoresce to report on protein unfolding as affected by amino acid substitutions and/or ligand binding. The WT MNV RdRp had an apparent *T*_m_ (*T*_Mapp_) of 43.5°C. R411A had a *T*_Mapp_ of ca. 2°C lower than that of the WT and the R416A mutant had a *T*_Mapp_ that was 1°C higher than that of the WT. Thus, both substitutions affected the conformation of the RdRp even before template binding.

The effects of Mps on the thermodenaturation profiles of the WT and mutant RdRps were also examined. However, no changes were observed in the *T*_Mapp_ with either the WT RdRp or the mutants. Mps did reduce the total fluorescence of the proteins, but the same effect was observed for the WT RdRp and the mutants (Figure 6G and H and data not shown). There was also a small change in fluorescence at ca. 55°C with R411A, the significance of which is as yet unknown (Figure 6H).

### Modification of GII.4 RdRp residues comparable to MNV RdRp R411 modestly increased RNA synthesis with Mps

**Figure 7.**
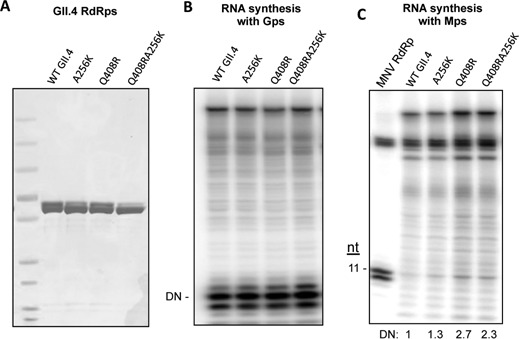
The GII.4 RdRp with a substitution of the residues Q408R and A256K which are comparable to R411 and K259 of the MNV RdRp had slightly improved RNA synthesis with the Mps. (**A**) WT and mutant GII.4 RdRps used in the assay. The gel image contains the purified proteins separated by SDS-PAGE and stained with Coomassie blue. The substitutions made in the GII.4 RdRp were to change the residues to those of present in the MNV RdRp. (**B**) RNA synthesis by the WT and mutant GII.4 RdRps using the Gps. All three mutant proteins had comparable activities for both de novo initiation and primer extension. (**C**) Gel image of the RNAs synthesized by the WT and mutant GII.4 RdRps using the Mps. The amount of the 11-nt RNA made by the various RdRps in this assay is quantified and listed below the gel image.

As we showed in Figure 1B, the GII.4 RdRp directed only low levels of *de novo* RNA synthesis from the Mps. Furthermore, the RdRp sequence alignment shows the GII.4 RdRp has a glutamine at position 408 and an alanine at position 256, which is comparable to R411 and K259 of the MNV RdRp (Figure 5E). These observations led us to determine whether GII.4 RdRp with substitutions of Q408R, A256K or both could improve *de novo* RNA synthesis with the heterologous MNV proscript. All three mutant RdRps produced comparable amounts of product from Gps when compared to the WT GII.4, indicating no loss of specificity for their cognate proscript (Figure 7A and B). In comparison to the WT MNV RdRp, the mutants directed much lower levels of *de novo* initiation of the 11-nt RNA from Mps. However, an approximately 2-fold increase was observed with the Q408R and the Q408R/A256K mutant proteins in comparison to the WT GII.4 RdRp (Figure 7C). Although the increase in RNA synthesis is modest, the results are reproducible in three independent assays. These results support the previous data suggesting that residue R411 in the MNV RdRp is important in directing *de novo*-initiated RNA synthesis by the MNV RdRp from the subgenomic core promoter. This data also suggests that GII.4 RdRp has additional requirements for the recognition of its subgenomic proscript than does the MNV RdRp.

### MNV virus production with mutations in the subgenomic promoter recognition

**Figure 8.**
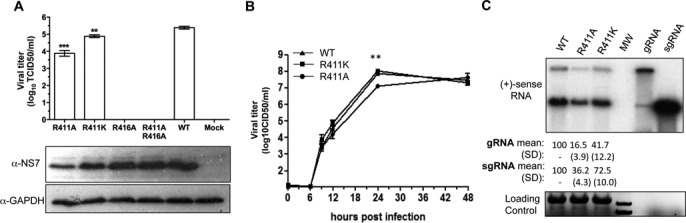
Mutations in the MNV RdRp that contacted the subgenomic promoter affect RNA synthesis during MNV infection. (**A**) Cell culture infectious dose for four viral constructs that have substitutions in the subgenomic promoter contact site. The bar graph summarizes the TCID_50_ for the mutant and WT viral constructs produced by BHK cells and titered in BV-2 cells. One Way ANOVA was used for significance testing, followed by a Dunnett post-test to compare all mutants to the WT. ***P < 0.001, **P < 0.01, *n* = 3. The lower two images are from western blots detecting the accumulation of the MNV RdRp, NS7 and the loading control, GAPDH. Note that mutant virus R416A and R411AR416A can express NS7, but that no viral progeny was produced. (**B**) Kinetics of viral progeny formation by the WT and mutant MNV. BV-2 cells were infected at a MOI of 1.0. The number of virion produced was determined by a limited dilution infection assay. The two asterisks (**) denote a *P* value of <0.01. (**C**) Accumulation of the genomic and subgenomic RNAs by the MNV mutants R411A and R411K in BV-2 cells. The upper image is from a northern blot of the total RNAs extracted 9 h after infection of BV-2 cells with mutant and the WT MNV at a MOI of 3. The lane labeled ‘MW’ contains a molecular weight ladder. Transcripts of the genomic RNA and sgRNA (the right-most two lanes) serve as molecular mass controls. The quantification of the genomic and sgRNAs are from three independent samples of the WT, R411A and R411K mutant viruses at 9 h after infection. The bottom image is of the ethidium bromide-stained denaturing agarose gel used for the northern blot. The bands are ribosomal RNAs and the MW standard. Transcripts of the gRNA and sgRNA are not present at an abundance to be detectable by ethidium bromide staining.

We sought to determine whether substitutions in the MNV RdRp that affected sgRNA initiation *in vitro* would affect MNV RNA replication in cells. Four infectious MNV cDNAs were engineered to express NS7 with alanine substitutions at R411 (R411A), a lysine substitution (R411K), an alanine substitution at R416 (R416A), or alanine substitutions of both R411 and R416 (R411AR416A). Each infectious clone was transfected into BHK cells, and cell lysates were collected. The TCID_50_ titer in the lysate was determined in BV-2 mouse microglial cells that can be infected by MNV. Mutants R411A and R411K had 10- and 3-fold lower viral titers than the WT MNV (Figure 8A). Sequencing of the R411A and R411K viruses revealed that they retain the engineered mutations and that they have no other mutations. In western blots, all four viruses produced the NS7 protein (Figure 8A). Constructs engineered to express the R416A and R411AR416A viruses failed to produce infectious virus (Figure 8A). Interestingly, the NS7 proteins were expressed in these BHK cells, indicating that residue R416 cannot be mutated and retain virion production (Figure 8A, bottom panel).

We characterized mutant viruses R411A and R411K for infection of BV-2 cells. The cells were infected with a 1 TCID_50_ of the WT and mutant viruses and the amount of virus released determined over a 48 h period (Figure 8B). Mutant R411A produced lower amounts of virus at the early time points, including a one-log decrease 24 h post infection (Figure 8B). At 9 h post-infection, mutants R411A and R411K had reduced levels of the genomic and sgRNAs, with the reduction being more severe with the R411A mutant (Figure 8C). Altogether, these results demonstrate that residue R411 identified in the MNV NS7 protein that contacts the hairpin of the MNV subgenomic promoter is important for MNV RNA production in the context of MNV infection.

## DISCUSSION

In this study, we expressed and purified recombinant MNV RdRp in *E. coli* to investigate the specific recognition of the MNV subgenomic promoter. Previous studies have identified a highly conserved RNA stem-loop structure as the core promoter for calicivirus sgRNA synthesis (24). However, it is unknown how the norovirus replication enzymes recognize the subgenomic promoter and facilitate RNA synthesis. We demonstrated that the recombinant RdRps from the GII.4 human norovirus and from MNV can specifically recognized their cognate subgenomic promoters and template sequences. Within the proscripts, both the hairpin and single-stranded regions that include the template sequence contribute to RNA synthesis. However, the GII.4 RdRp displayed higher specificity for its own subgenomic promoter hairpin and template sequence than did the MNV RdRp, which was also able to initiate RNA synthesis from the GII.4 proscript, albeit with lower efficiency.

The residues in the MNV RdRp that contact the subgenomic promoter hairpin were mapped (Figure 5). Mutations in several residues found to contact the hairpin resulted in RdRps that were defective for RNA synthesis. Amino acid residues R411 and R416 were found to contribute to *de novo* initiated RNA synthesis and the affinity of RNA binding. The two residues in the NS7 protein are also required for wild-type levels of MNV RNA synthesis in the context of MNV infection. Notably, substitutions at R411 were less detrimental for RNA synthesis *in vitro* and mutant virus with substitutions at residue R411 retained infectivity in cells (Figure 8). Mutant MNV with the R411A substitution had decreased subgenomic and genomic RNA synthesis (Figure 8C). It is possible that the same residues participate in additional modes of RNA synthesis; hence there were significant decreases in the levels of genomic RNA.

Three regions of the MBV RdRp were identified to contact the hairpin within the MNV subgenomic promoter: a sequence near motif F, residues near motif A within the finger subdomain, and the α-helix near motif E. Lee *et al.* found that residue R185 near motif F was involved in binding to the RNA in all available viral RdRp structures including distantly related HIV reverse transcriptase (39). The F1 motif of the Dengue virus RdRp was also shown to be involved in promoter-dependent *de novo* RNA synthesis (48). For the MNV RdRp, a R185A substitution resulted in a severely defective for both primer-extension and *de novo* initiated RNA synthesis. Mutant RdRp R255A near motif A also showed defective polymerase activity for both primer extension and *de novo* initiated RNA synthesis. It is likely that R185 and R255 serve to stabilize core promoter binding.

Mutations in the α-helix near motif E had more complex effects on RNA synthesis. Mutant R411A was specifically defective for *de novo* initiation but fully competent for primer extension. Mutant R416A was defective for *de novo* initiation but also for the synthesis of the full-length product, likely due to an effect the termination of RNA synthesis. R411A and R416A both had similar defect in binding Mps. It is likely that R411 and R416 maintain contact with the proscript and the template throughout the process of RNA synthesis.

Our results show that the norovirus RdRp can specifically recognize the subgenomic promoter and the initiation cytidylate in the absence of other viral proteins. This was somewhat surprising given that the norovirus sgRNA is likely to be VPg-linked to facilitate translation (49). Addition of recombinant VPg to the *in vitro* RNA synthesis assays did not result in a significant amount of protein-primed product, possibly due to the lack of RNA elements that will promote the formation of the VPg-nucleotide complex (Lin, data not shown; 50). Furthermore, VPg nucleotidylation prefers GTP, and it is possible that GTP and VPg-GMP are functionally equivalent in triggering RNA synthesis.

The norovirus subgenomic promoter consists of two components: the RNA hairpin and the template sequence. The hairpin motif has been previously demonstrated to serve as the promoter for RNA synthesis by plant viruses (51–53). In the noroviruses, the hairpin can specifically contact the noroviral RdRp, likely to position the RdRp in the proper context for the initiation of RNA synthesis. In addition to the RNA hairpin motif, our results also show the single-stranded sequence that contains the template initiation nucleotide can significantly influence proscript recognition (Figure 2C). There is also precedent for unstructured sequence modulating the level of RNA synthesis (30,46,54–55). Butcher *et al.* (56) have identified various specific contacts between the template RNA and the phage ϕ6 RdRp during *de novo* initiated RNA synthesis. This level of specificity for the template sequence could prevent the viral replication complex from misrecognition of the RNAs which could lead to defects in the process of RNA synthesis and possibly increase the detection of the RNA products by innate immune receptors (57).

RdRps are one of the four major classes of template-dependent polymerases that also include DNA-dependent DNA polymerases, the reverse transcriptases, and the DNA-dependent RNA polymerases. It is worth noting that core promoter recognition by the recombinant norovirus RdRps is a feature shared with bacteriophage DNA-dependent RNA polymerases (37,58–59). Iyer *et al*. (60) had proposed that the ancestor of the RNA polymerases contains an RNA-binding domain that preceded the acquisition of polymerase activity. Higher resolution analysis of the interactions between the norovirus RdRps and their cognate promoters would therefore provide insight to potentially ancient protein–RNA interaction as well as inform the mechanism of viral RNA synthesis.

## SUPPLEMENTARY DATA

Supplementary Data are available at NAR Online.

SUPPLEMENTARY DATA
