# Supplementary material for: Subgenomic promoter recognition by the norovirus RNA-dependent RNA polymerases
Source: Nucleic Acids Res. 2014 Dec 17;43(1):446–60. doi: 10.1093/nar/gku1292 (PMC4288183; doi:10.1093/nar/gku1292)
Supplement: SUPPLEMENTARY DATA [file supp_43_1_446__index.html]

Subgenomic promoter recognition by the norovirus RNA-dependent RNA polymerases — Subgenomic promoter recognition by the norovirus RNA-dependent RNA polymerases — SUPPLEMENTARY DATA 

# Subgenomic promoter recognition by the norovirus RNA-dependent RNA polymerases

## SUPPLEMENTARY DATA

**Files in this Data Supplement:**

- SUPPLEMENTARY DATA
