# Supplementary material for: Subgenomic promoter recognition by the norovirus RNA-dependent RNA polymerases
Source: Nucleic Acids Res. 2014 Dec 17;43(1):446–60. doi: 10.1093/nar/gku1292 (PMC4288183; doi:10.1093/nar/gku1292)
Supplement: SUPPLEMENTARY DATA [file supp_gku1292_nar-02323-v-2014-File010.pptx]

## Slide 1
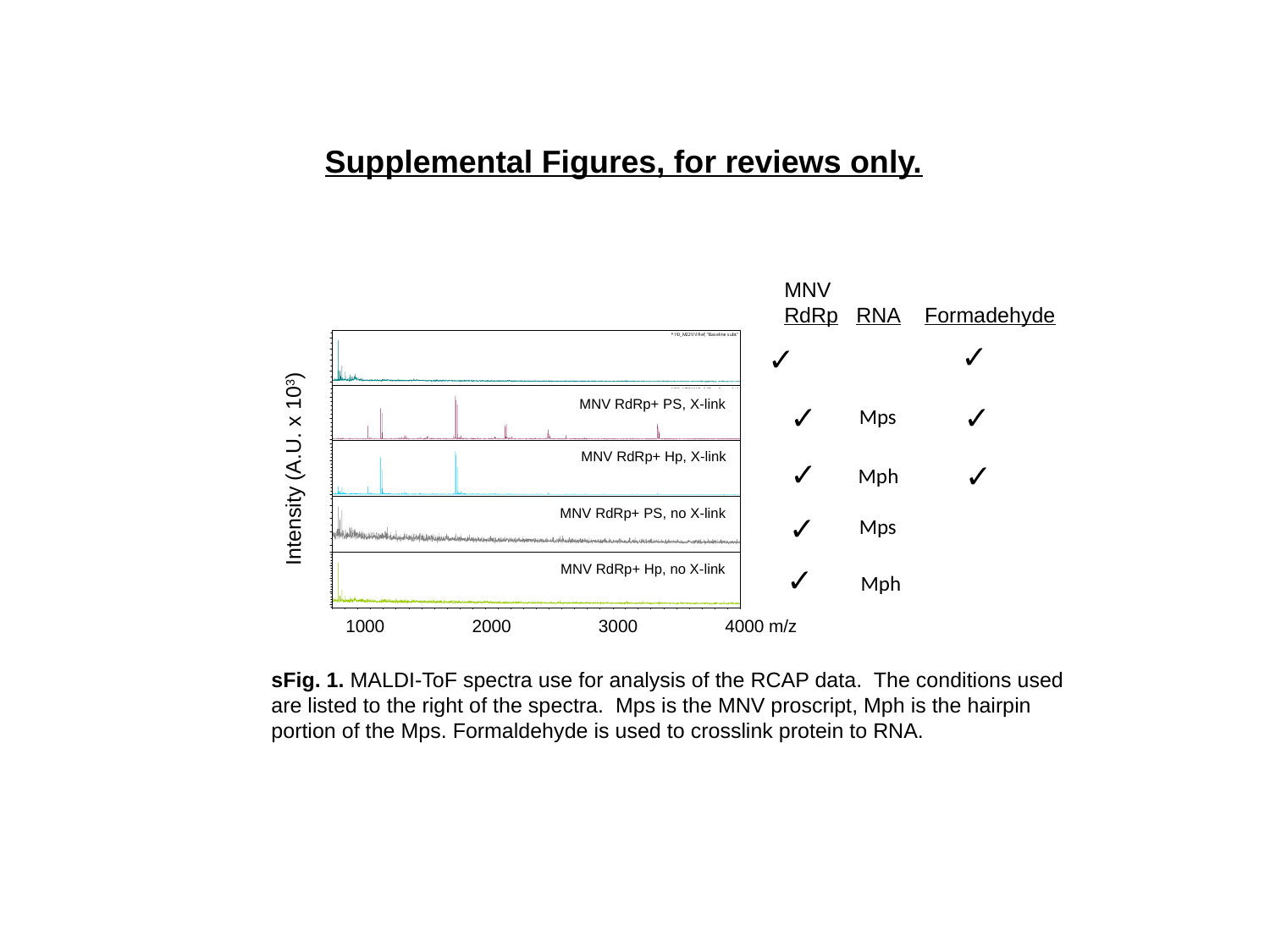

Supplemental Figures, for reviews only.
MNV
RdRp RNA Formadehyde
✓
✓
MNV RdRp+ PS, X-link
✓
✓
Mps
MNV RdRp+ Hp, X-link
✓
Intensity (A.U. x 103)
✓
Mph
MNV RdRp+ PS, no X-link
✓
Mps
MNV RdRp+ Hp, no X-link
✓
Mph
1000 2000 3000 4000 m/z
sFig. 1. MALDI-ToF spectra use for analysis of the RCAP data. The conditions used are listed to the right of the spectra. Mps is the MNV proscript, Mph is the hairpin portion of the Mps. Formaldehyde is used to crosslink protein to RNA.

## Slide 2
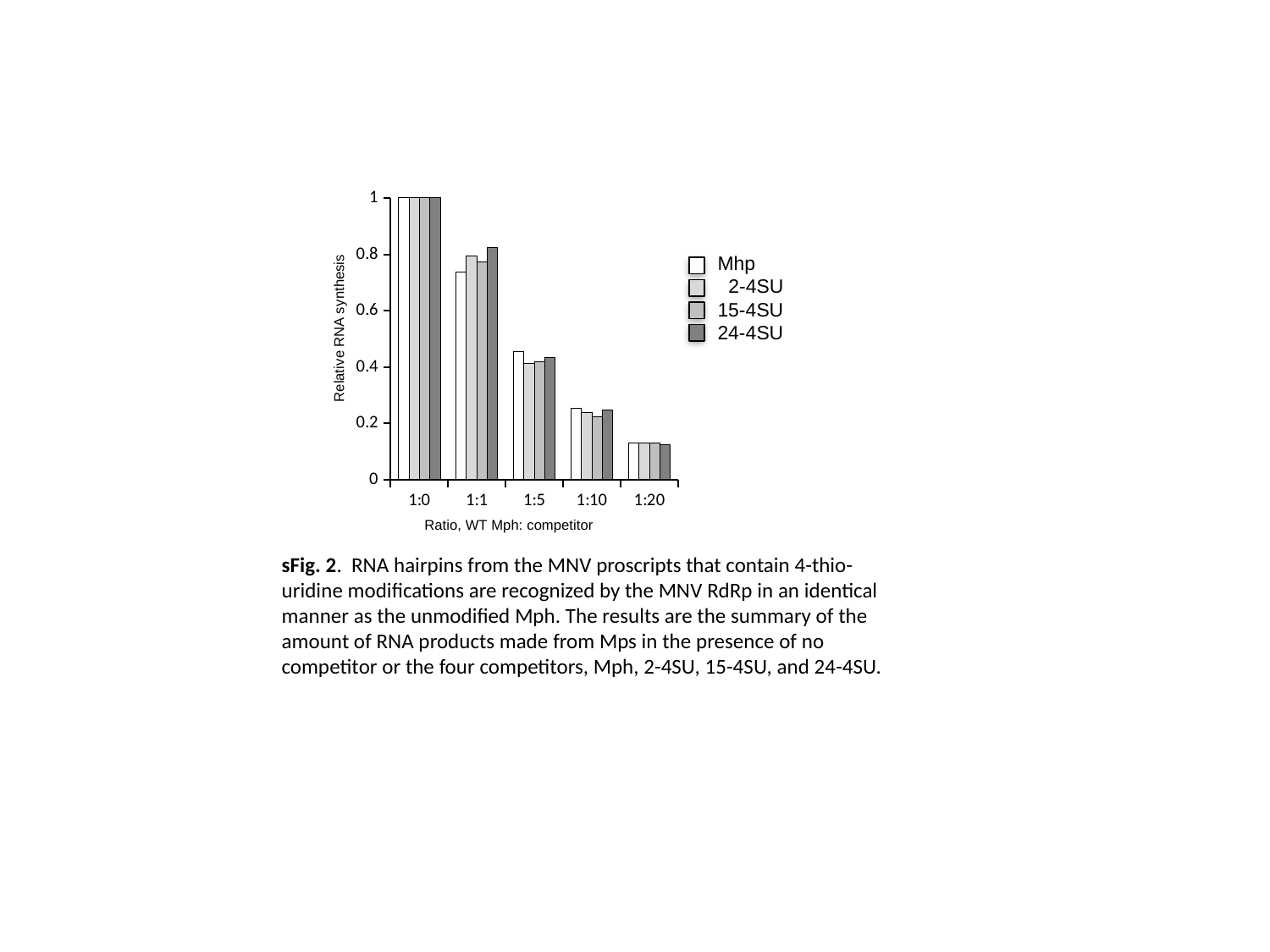

### Chart
| Category | Ps/Hp | Ps/Hp-2SU | Ps/Hp-15SU | Ps/Hp-24SU |
|---|---|---|---|---|
| 1:0 | 1.0 | 1.0 | 1.0 | 1.0 |
| 1:1 | 0.736540370608741 | 0.794452574333424 | 0.773310689281351 | 0.823544919811832 |
| 1:5 | 0.453730803333635 | 0.411221489988411 | 0.419413236818772 | 0.433309106807982 |
| 1:10 | 0.252687920752344 | 0.238010357859225 | 0.221919866484928 | 0.246438537631463 |
| 1:20 | 0.131652689511383 | 0.129461779597351 | 0.129836393461612 | 0.124848257933371 |Mhp
 2-4SU
15-4SU
24-4SU
Relative RNA synthesis
Ratio, WT Mph: competitor
sFig. 2. RNA hairpins from the MNV proscripts that contain 4-thio-uridine modifications are recognized by the MNV RdRp in an identical manner as the unmodified Mph. The results are the summary of the amount of RNA products made from Mps in the presence of no competitor or the four competitors, Mph, 2-4SU, 15-4SU, and 24-4SU.

## Slide 3
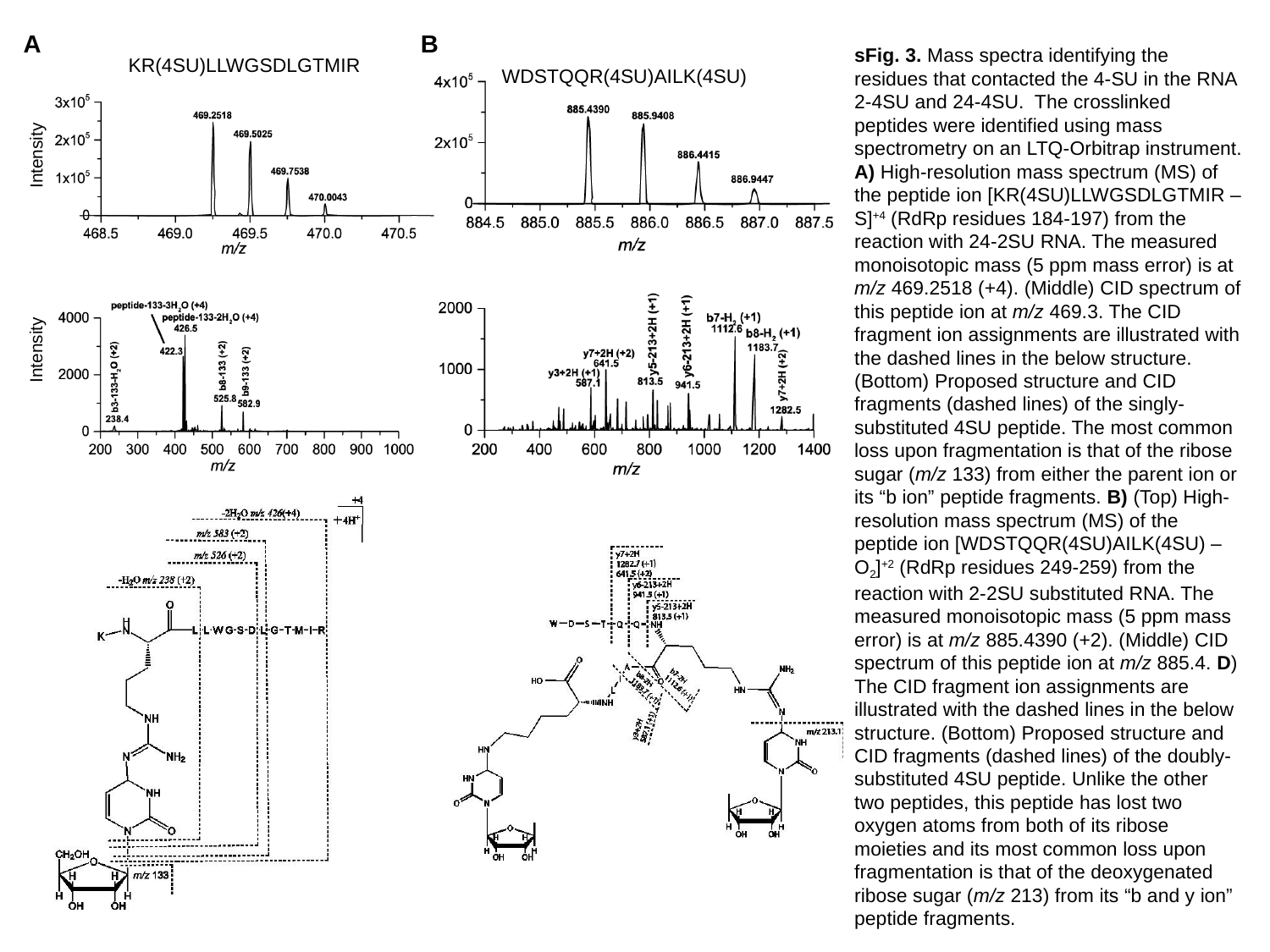

A
B
sFig. 3. Mass spectra identifying the residues that contacted the 4-SU in the RNA 2-4SU and 24-4SU. The crosslinked peptides were identified using mass spectrometry on an LTQ-Orbitrap instrument. A) High-resolution mass spectrum (MS) of the peptide ion [KR(4SU)LLWGSDLGTMIR – S]+4 (RdRp residues 184-197) from the reaction with 24-2SU RNA. The measured monoisotopic mass (5 ppm mass error) is at m/z 469.2518 (+4). (Middle) CID spectrum of this peptide ion at m/z 469.3. The CID fragment ion assignments are illustrated with the dashed lines in the below structure. (Bottom) Proposed structure and CID fragments (dashed lines) of the singly-substituted 4SU peptide. The most common loss upon fragmentation is that of the ribose sugar (m/z 133) from either the parent ion or its “b ion” peptide fragments. B) (Top) High-resolution mass spectrum (MS) of the peptide ion [WDSTQQR(4SU)AILK(4SU) – O2]+2 (RdRp residues 249-259) from the reaction with 2-2SU substituted RNA. The measured monoisotopic mass (5 ppm mass error) is at m/z 885.4390 (+2). (Middle) CID spectrum of this peptide ion at m/z 885.4. D) The CID fragment ion assignments are illustrated with the dashed lines in the below structure. (Bottom) Proposed structure and CID fragments (dashed lines) of the doubly-substituted 4SU peptide. Unlike the other two peptides, this peptide has lost two oxygen atoms from both of its ribose moieties and its most common loss upon fragmentation is that of the deoxygenated ribose sugar (m/z 213) from its “b and y ion” peptide fragments.
KR(4SU)LLWGSDLGTMIR
WDSTQQR(4SU)AILK(4SU)
Intensity
Intensity

## Slide 4
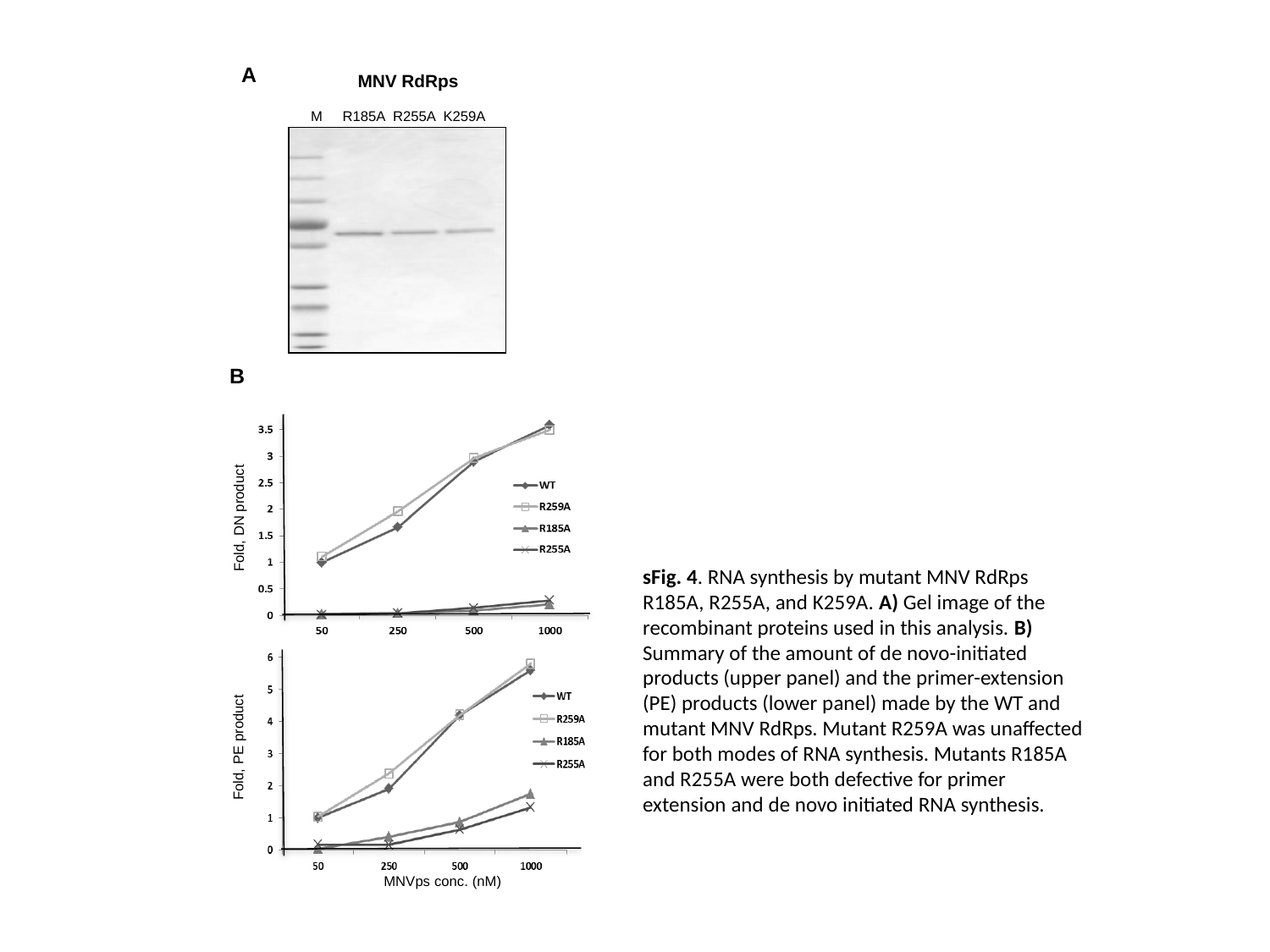

A
MNV RdRps
M R185A R255A K259A
B
Fold, DN product
sFig. 4. RNA synthesis by mutant MNV RdRps R185A, R255A, and K259A. A) Gel image of the recombinant proteins used in this analysis. B) Summary of the amount of de novo-initiated products (upper panel) and the primer-extension (PE) products (lower panel) made by the WT and mutant MNV RdRps. Mutant R259A was unaffected for both modes of RNA synthesis. Mutants R185A and R255A were both defective for primer extension and de novo initiated RNA synthesis.
Fold, PE product
MNVps conc. (nM)
